# Annual Incidence of Dementia from 2003 to 2018 in Metropolitan Seoul, Korea: A Population-Based Study

**DOI:** 10.3390/jcm11030819

**Published:** 2022-02-03

**Authors:** Hyuk Sung Kwon, Yong Whi Jeong, Seung Hyun Kim, Kee Hyung Park, Sang Won Seo, Hae Ri Na, Seong-Ho Koh, YongSoo Shim, Moon Ho Park, Juhee Chin, Sojeong Park, Dae Ryong Kang, Hojin Choi

**Affiliations:** 1 Department of Neurology, Hanyang University Guri Hospital, Hanyang University College of Medicine, Guri 11923, Korea; kwonhs@hanyang.ac.kr (H.S.K.); ksh213@hanyang.ac.kr (S.-H.K.); 2 Department of Information and Statistics, Yonsei University, Wonju 26493, Korea; wjddydgnl7@naver.com; 3 Department of Neurology, Hanyang University College of Medicine, Seoul 04763, Korea; kimsh1@hanyang.ac.kr; 4 Department of Neurology, College of Medicine, Gachon University Gil Hospital, Incheon 21565, Korea; khpark0813@gmail.com; 5 Department of Neurology, Samsung Medical Center, Sungkyunkwan University School of Medicine, Seoul 06351, Korea; sangwonseo@empal.com (S.W.S.); juheechin@hanmail.net (J.C.); 6 Department of Neurology, Bobath Memorial Hospital, Seongnam 13552, Korea; neuna102@gmail.com; 7 Department of Neurology, Eunpyeong St. Mary’s Hospital, College of Medicine, The Catholic University of Korea, Seoul 03312, Korea; ysshim@catholic.ac.kr; 8 Department of Neurology, Korea University Ansan Hospital, Ansan 15355, Korea; kmmse@naver.com; 9 Data Science Team, Hanmi Pharm. Co., Ltd., Seoul 05545, Korea; sojeong.park@hanmi.co.kr; 10 Department of Biostatistics, Yonsei University Wonju College of Medicine, Wonju 26426, Korea; dr.kang@yonsei.ac.kr

**Keywords:** dementia, incidence, dementia support center, national health insurance service, policy

## Abstract

National dementia plans were applied in dementia support centers established in Seoul, Korea between 2007 and 2009. However, the annual incidence rates of dementia in Seoul have not been reported. We investigated this annual incidence and the characteristics of incident cases from 2003 to 2018. The customized research database of the Korean National Health Insurance Services was used. The annual crude and age-standardized incidence of dementia patients and their characteristics were analyzed. This study analyzed 108,596 incident dementia cases aged ≥60 years. The incidence rate increased from 2003 to 2011, including a rapid increment from 2007 to 2011. From 2011 to 2018, the crude (age-standardized) incidence per 10^5^ person-years decreased from 641.51 (577.12) to 448.26 (361.23). The proportion of incident dementia cases was highest in the highest income group every year. However, the proportion of incident dementia cases in the lowest income group increased from 10.4% in 2003 to 25.8% in 2011. The annual incidence rate of dementia showed a sharp increase immediately after 2007, the year dementia support centers began to be introduced, and then stabilized after 2011. The characteristics of incident dementia cases have changed, including the proportion in the low-income group.

## 1. Introduction

Dementia is a clinical syndrome of cognitive decline that causes interference in social or occupational functioning [1]. There were around 50 million people worldwide with dementia in 2018, and this number is estimated to triple by 2050 with the aging of the population [2]. Although some studies have reported a decline in the prevalence and incidence of dementia in high-income countries [3,4,5], the majority of the population that could affect the increment of dementia live in low- to middle-income countries [2,6]. Considering the socioeconomic burden caused by dementia, many countries are making efforts to manage the incidence of dementia.

In Korea, there was a declaration of a “war on dementia” in 2008 [7]. Beginning with 4 dementia support centers (DSCs) in 2007, a total of 25 DSCs were established in Seoul, the capital and largest metropolis of Korea, through 2009. In addition, national dementia plans (NDPs) were released in 2018 and conducted with DSCs. The purposes of DSCs and NDPs are to find dementia patients in earlier stages with dementia screening programs, to prevent dementia by managing risk factors, to improve awareness of dementia, and to reduce the diverse burdens on dementia patients and their caregivers [8]. As a result of the diverse policies, including the dementia screening program, we expected that the incidence of dementia would increase rapidly but temporarily.

It is important to obtain and respond to estimated epidemiological data to evaluate the effects of policies and design new policies. We focused our attention on Seoul, as DSCs were established intensively in all 25 districts of Seoul from 2007 to 2009, and NDPs were carried out. The annual incidence of dementia in Seoul has not been reported previously. Therefore, in this study, we investigated how the incidence of dementia in Seoul changed before and after the establishment of DSCs using customized research data extracted from the National Health Insurance (NHIS) Database. In addition, we identified the characteristics of newly diagnosed dementia patients from 2003 to 2018.

## 2. Materials and Methods

### 2.1. Data Source

This study was conducted using anonymous customized research data extracted from the NHIS Database between 1 January 2002 and 31 December 2018. This database is based primarily on the Korean NHIS, a single government insurer that covers about 97% of the Korean population. Korean hospitals and nursing facilities are supported by the NHIS. The customized database is representative of the transmission data provided by anonymous health insurance and long-term care insurance data [9]. The database provides healthcare utilization information for both inpatients and outpatients and includes patient demographics, diagnosis, comorbidities, and prescribed medication. The Korean Classification of Disease (KCD), 5th to 7th editions, and modification of the International Classification of Disease and Related Health Problems, 10th revision were used to code diagnoses. Data on demographics (including age, sex, and income), accompanying diagnostic codes including diabetes (E10~14), chronic obstructive pulmonary disease (J44), chronic kidney disease (N18), dyslipidemia (E78), stroke (I60~64), hypertension (I10~15), and depression (F32, F33, F34.1) were collected using the NHIS coding system. The type of antidementia drug (donepezil, galantamine, rivastigmine, or memantine) was collected, and pharmaceutical prescription codes for antidementia drugs are described in Table S1 (Supplementary Materials).

### 2.2. Ethical Approval

This study was approved by the Institutional Review Board of Hanyang University Guri Hospital (2019–10-004) and registered at the Clinical Research Information Service (CRIS) under the registration number KCT0006243. All personal information in the NHIS database was de-identified, and informed consent was waived.

### 2.3. Study Population

**Figure 1:**
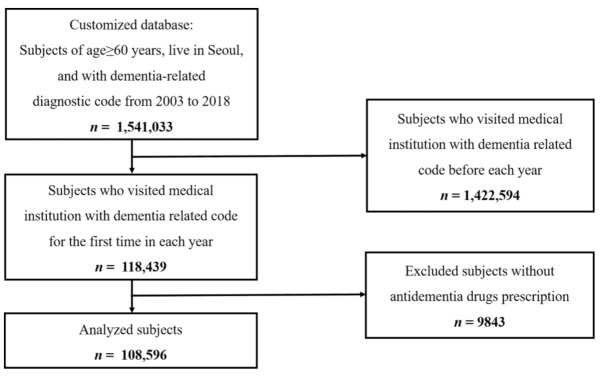
Identification of newly diagnosed dementia patients aged ≥60 in Seoul from the National Health Insurance Database.

All individuals in the current customized research database were aged 60 or more, registered in Seoul, and visited a medical institution with a recorded dementia-related diagnostic code from 2003 to 2018 (Figure 1). Dementia was identified in the claims data based on the KCD-5, -6, or -7 code. Dementia patients were defined as those who had a history of outpatient visits or admissions with a dementia-related diagnostic code and who used antidementia drugs. Dementia-related diagnostic codes were F00 (Dementia in Alzheimer’s disease), F01 (Vascular dementia), F02 (Dementia in other diseases classified elsewhere), F03 (Unspecified dementia), G30 (Alzheimer’s disease), G31.00 (Behavioral variant frontotemporal dementia), G31.01 (Semantic variant primary progressive aphasia), G31.02 (Nonfluent primary progressive aphasia), G31.03 (Logopenic primary progressive aphasia), G31.04 (Primary progressive aphasia), and G31.82 (Dementia with Lewy bodies). Those with a record of claims data with a dementia-related diagnostic code in 2002 or earlier were excluded.

### 2.4. Statistical Analysis

The annual number of dementia patients in Seoul was identified. All participants were divided according to age (5 groups: 60–64, 65–69, 70–74, 75–79, 80–84, and ≥85), sex, and income (quintiles) and compared. Continuous variables are expressed as mean ± SD, and categorical variables are expressed as percentage or frequency. The crude incidence stratified by age and sex was calculated. In addition, age-adjusted incidence using the standard population (Seoul population covered by health insurance in 2003) in Seoul was calculated to compare years. All statistical analyses were performed using the SAS system version 9.4 (SAS Institute Inc., Cary, NC, USA), and *p* < 0.05 was considered statistically significant.

## 3. Results

Between 2003 and 2018, a total of 1,541,033 cases aged ≥60 years had at least 1 healthcare visit with a dementia-related diagnostic code in Seoul. Among these, there were 118,439 incident cases. Finally, 108,596 cases were prescribed antidementia medications and analyzed in the current study (Figure 1). The overall incidence rate per 10^5^ person-years from 2003 to 2018 was 457.23 (95% CI: 454.51–459.95) and was higher in females (551.59 (95% CI: 547.54–555.64)) than males (344.84 (95% CI: 341.35–348.34)).

**Figure 2:**
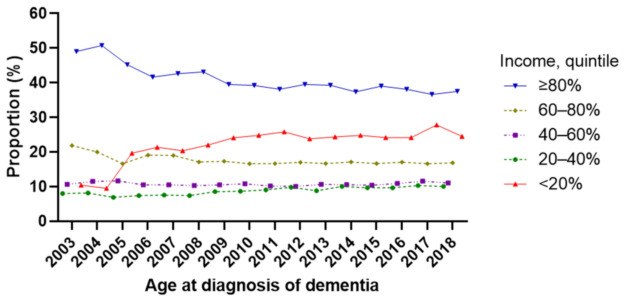
Proportion of newly diagnosed dementia patients according to income.

The overall demographics, comorbidities, and prescribed medications of the incident cases are described in Table 1. The number of incident cases increased with age and was higher in females. Among the incident dementia cases, 19.7% had hypertension, 8.0% had diabetes, 9.1% had dyslipidemia, and 15.0% had depression-related diagnostic codes. Donepezil (74.9%) was the most prescribed drug at dementia diagnosis, followed by rivastigmine (9.7%), memantine (9.0%), and galantamine (6.4%). Demographics, comorbidities, and prescribed medications of the incident cases in each year are described in Table 2 (from 2003 to 2010) and Table 3 (from 2011 to 2018). Among comorbidities, the percentage of dementia cases with hypertension decreased gradually since 2006, while those with dyslipidemia increased steadily since 2003. The proportion of incident dementia cases was highest in the highest income group (≥80%) every year. However, the proportion of incident dementia cases in the lowest income group (<20%) increased from 10.4% in 2003 to 25.8% in 2011 and has remained at that level (Figure 2).

**Figure 3:**
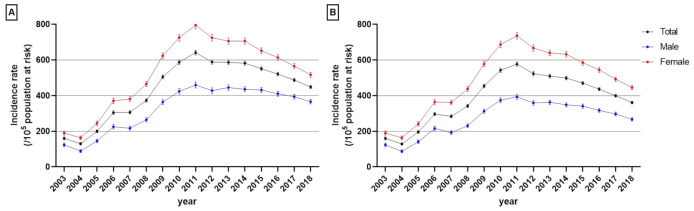
Annual crude (**A**) and age-standardized (**B**) incidence of dementia stratified by sex. Error bars indicate the 95% confidence interval.

The crude incidence of dementia per 100,000 population aged ≥60 years in Seoul increased gradually from 129.82 (95% CI: 122.87–136.66) in 2004 to 641.51 (95% CI: 628.44–654.59) in 2011 and included a rapid increment between 2007 and 2011 (Figure 3 and Table S2, Supplementary Materials). After 2011, the crude incidence decreased gradually to 448.26 (985% CI: 438.90–457.61) in 2018 (Figure 3A and Table S2, Supplementary Materials). The age-standardized incidence of dementia showed a similar pattern, with an incidence per 10^5^ subjects aged ≥60 years which increased from 160.27 (152.50–168.04) in 2003 to 283.54 (275.01–292.07) in 2007 and peaked in 2011 at 577.12 (95% CI: 565.29–588.96). The rate then decreased gradually to 361.23 (95% CI: 353.58–368.88) in 2018 (Figure 3B).

**Figure 4:**
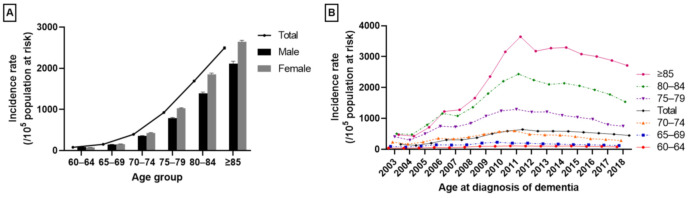
The incidence of dementia stratified by age. (**A**) Crude incidence data of the whole group from 2003 to 2018. (**B**) Annual incidence of dementia stratified by age group. Error bars indicate the 95% confidence interval.

The incidence rates of dementia in the whole group increased with age (Figure 4A,B) from 82.01 per 10^5^ population at risk (95% CI: 80.05–83.98) at the age of 60–64 years to 2496.09 (95% CI: 2464.93–5527.25) at age ≥85 years. The incidence rate was slightly higher in males aged 60–64 years; in the other age groups, it was higher in females (Figure 4A). The annual incidence rates of dementia patients stratified by age group are shown in Figure 4B.

## 4. Discussion

This study found that the incidence of all-cause dementia in the Seoul population aged 60 years or older increased from 2003 (160.3/10^5^ person-years) to 2007 (305.9/10^5^ person-years). There was a more rapid increase from 2008 (373.4/10^5^ person-years) to 2011 (641.5/10^5^ person-years) and a gradual decrease from 2011 to 2018 (448.3/10^5^ person-years). The incidence rate was higher in females across all ages except 60–64 years and increased with age. In addition, demographics, income, comorbidities, and prescribed antidementia medications were considered. The proportion of incident dementia cases within the lowest income group steadily increased from 2007 to 2011.

Incidence rates in the current study (4.6/10^3^ person-years) are comparable with those from other countries, although they are difficult to compare directly [10,11]. Incidence rates of all-cause dementia and Alzheimer’s disease (AD) differ considerably among previous reports: 2.3~65.6/10^3^ person-years (all-cause dementia) and 0.04~16.8/10^3^ person-years (AD), respectively [10,11]. These differences might be due to variations in data collection procedures, age ranges, follow-up schedules, and diagnostic criteria [6,12]. In addition, different genetic and environmental factors can contribute to differences. Therefore, we focused on trends in the change of incidence rates rather than exact incidence rates. 

In Europe and the United States, the incidence rate of dementia is declining [5]. In contrast, the incidence of dementia is increasing in Taiwan [12], Beijing [13], and Japan [14]. In Korea, one study reported a decreasing trend of dementia incidence by comparing two rural cohort studies (individuals in 1996 vs. 2008) targeting the population in Yeoncheon county [15]. Another study analyzed the NHIS senior cohort (representing 10% of the population aged ≥60) that was sampled in 2002 and showed an increment in the standardized incidence of dementia from 2003 to 2015 in Korea [16]. However, as there was no influx of new cases after 2002 in the study using the NHIS senior cohort, this increasing trend of incidence might be due to the aging of the target population. In the current study, dementia patients were selected from the entire Seoul population each year using the same criteria. To our knowledge, our study is the first to estimate the annual incidence of dementia in the Seoul population.

In Korea, 25 DSCs were established in the districts of Seoul between 2007 and 2009. DSCs carried out NDPs, including establishing a dementia screening program, managing risk factors to prevent dementia, improving awareness of dementia, and supporting dementia patients and caregivers to reduce socioeconomic burden. Three NDPs were announced during the study period: NDP-1 (2008 to 2011), NDP-2 (2012 to 2015), and NDP-3 (2016 to 2020). An introduction of these NDPs have been previously provided [17]. It is hard to divide the objectives and strategies of these three NDPs clearly. NDP-1 was focused on the early diagnosis of cognitive impairment, delaying its progression and installing DSCs to establish a community-based dementia management system. NDP-2 included expanding dementia management programs (i.e., dissemination of the 3-3-3 rule to prevent dementia, developing apps for dementia self-checkup), expanding socioeconomic support (i.e., expanding eligibility for long-term care insurance for dementia patients under the name of “5th Grade”), expansion of infrastructures (i.e., training dementia specialists with education programs, supporting dementia research, and establishing the dementia management conveyance system), and improving awareness of dementia (i.e., installation of the “national dementia helpline”, implementation of “annual dementia awareness day”, and operation of dementia partner programs). NDP-3 tried to enhance community-based prevention and management of dementia, care, and treatment for dementia patients, reduce care burden for caregivers, and support through research, statistics, and technology. The main objectives of NDP-3 were the expansion of the dementia partner program, initiation of dementia-friendly communities, and intensive management for dementia high-risk groups [8,17]. These policies were designed to identify dementia patients who were not receiving medical care. A rapid increment of incident dementia cases in the NHIS database from 2007 to 2011 might be a result of these policies. However, such an increment has not been observed since 2011. Previous reports have raised concerns about dementia policies to screen for early-stage dementia due to the rapid increase in dementia patients and overdiagnosis [18,19]. However, according to the results of our study, the rapid increase in the incidence rate was temporary after initiation of the dementia screening programs and began to decrease about three years later. In Korea, dementia screening programs by DSCs have been implemented nationwide since 2017. A similar trend is expected in the nationwide dementia incidence rate. As the basic reference for establishing dementia policies is epidemiologic data, the results of the current study are expected to serve as evidence for future dementia policies.

In all years, the proportion of incident dementia cases was highest in individuals within the highest income group (top 20%). Although previous reports show an association between dementia and lower socioeconomic status [20,21], high-income individuals perhaps are more aware of their cognitive change, continue to consult their physicians and take cognitive function tests, and receive an earlier diagnosis [22]. However, the proportion of incident dementia in individuals within the lowest income group (bottom 20%) increased steadily from 20.4% in 2007 to 25.8% in 2011 (Table 2 and Table 3, and Figure 2). Dementia might be under-recognized and under-disclosed in low-income individuals. Therefore, the observed increase might be the result of efforts to improve awareness of dementia among low-income individuals and to create an environment where it is easier to receive cost-free dementia screening.

Diverse factors including diabetes, hypertension, and depression are known to be associated with increased risk of dementia and cognitive decline [23,24,25,26]. Although the role of cholesterol in dementia remains unclear, previous reports have demonstrated that high total cholesterol level is a risk factor for dementia [27]. The prevalence of diabetes, hypertension, dyslipidemia, and depression are increasing in Korea [28,29], showing a plateaued control rate. In this study, dementia patients with diabetes and hypertension showed a decrease from 2011 and 2006, respectively. This decrement might be another reason for the gradual reduction of dementia incidence from 2011 in Seoul, although dementia patients with depression increased beginning in 2014. In addition, as educating the importance of managing vascular risk factors to patients with mild cognitive impairment plays another role of DSCs, regulation of blood pressure, blood pressure variability, diabetes, glycemic variability, and dyslipidemia might have contributed to the incidence of dementia [30,31,32]. We additionally compared the characteristic of incident dementia patients before and after 2008, the year most DSCs were introduced and performed dementia screening programs (Table S3, Supplementary Material). Although dementia patients with diabetes were higher, most of the comorbidities, including stroke, hypertension, depression, peptic ulcer, and congestive heart failure, were lower in patients who were diagnosed with dementia after 2008 than before 2008. The proportion of incident dementia cases within the lowest income group and the highest age group were higher in patients who were diagnosed with dementia after 2008.

There are some limitations. First, we identified dementia patients using diagnostic codes and the use of antidementia medications from NHIS databases; the diagnosis could not be double-checked with neuropsychological tests. Identifying the results of the neuropsychological tests, including interviews and questionnaires, can be helpful. However, as door-to-door interview surveys or questionnaire-based studies require high effort and costs, it is difficult to estimate the incidence for every year. Using electronic NHIS databases allows the study of the epidemiology of dementia every year and the determination of the effect or direction of dementia policies. Second, we have tried to reduce overestimation by limiting this study to cases with a dementia-related diagnostic code and prescribed antidementia medications, as in previous reports [16,33]. However, some individuals who were not receiving antidementia medications, especially patients with dementia other than Alzheimer’s disease, or not visiting medical institutions might have been excluded. However, among the 118.349 patients who initially visited medical institutions with dementia-related diagnostic codes, only 9843 (8.3%) did not take antidementia medications. Third, different subtypes of dementia were not addressed, as it was outside the scope of the current study. Future studies should investigate the proportion and change of dementia subtypes. Fourth, comorbidities such as cancer were not analyzed in the current study. The mechanism between cancer and neurodegenerative disease is unclear, such that some reports showed a positive relationship between cancer and α-synucleinopathy [34,35], while other reports demonstrated an inverse correlation between cancer and dementia [36]. Future studies should consider cancer as comorbidities while analyzing the epidemiology of dementia. Fifth, the analysis of the current study was limited to the Seoul population. As DSCs were conducted in all districts of Seoul first, we focused on the incidence of dementia in Seoul, the largest city of Korea. A similar trend is expected for the nationwide dementia incidence rate, so it is important to know the trend of incidence in the Seoul population. Additionally, predicting changes in the incidence is important for preparing new policies with limited resources. Future studies analyzing the dementia incidence in a nationwide population or comparing Seoul and other regions are needed to demonstrate the further effectiveness of NDPs. Sixth, the annual cost per dementia patient and compliance to medications were not analyzed. Further studies with this information might provide more detailed results.

## 5. Conclusions

Based on the customized NHIS database, incidence rates of dementia in those aged ≥60 years in Seoul increased from 2004 to 2011 and included a marked increment between 2007 and 2011. A decrease occurred from 2011 to 2018. Incidence rates were higher in females and with older age. Among incident dementia cases, the proportion of the lowest income group increased from 2007 to 2011, and the proportion with comorbidities changed each year. We anticipate that these results will serve as evidence for establishing future dementia policies.

## Figures and Tables

**Table 1:** Demographic characteristics and comorbidities of dementia patients who were diagnosed with dementia from 2003 to 2018.

Total Number	108,596 (100)
Age	
60~64	6674 (6.1)
65~69	9393 (8.7)
70~74	17,064 (15.7)
75~79	35,382 (32.6)
80~84	25,429 (23.4)
≥85	24,654 (22.70)
Sex, female	71,214 (65.6)
Income, quintile	
<20%	25,378 (23.4)
20~40%	9971 (9.2)
40~60%	60,784 (11.0)
60~80%	89,841 (16.4)
≥80%	168,756 (30.7)
Comorbidities	
Diabetes	8649 (8.0)
COPD	334 (0.3)
CKD	545 (0.5)
Dyslipidemia	9857 (9.1)
Stroke	25,723 (23.7)
Hypertension	21,403 (19.7)
Depression	16,319 (15.0)
Antidementia medication	
Donepezil	81,385 (74.9)
Galantamine	6933 (6.4)
Rivastigmine	10,486 (9.7)
Memantine	9792 (9.0)

Data are presented as mean ± SD, number (%), unless otherwise indicated.

**Table 2:** Characteristics of dementia patients according to year of diagnosis (2003~2010).

	2003	2004	2005	2006	2007	2008	2009	2010
Total number	1634 (100)	1385 (100)	2216 (100)	3484 (100)	4280 (100)	5496 (100)	7810 (100)	8233 (100)
Age								
60~64	165 (10.1)	146 (10.5)	169 (7.6)	261 (7.5)	240 (5.6)	331 (6.0)	495 (6.3)	480 (5.8)
65~69	250 (15.3)	205 (14.8)	300 (13.5)	455 (13.1)	550 (12.9)	598 (10.9)	838 (10.7)	852 (10.4)
70~74	359 (22.0)	279 (20.1)	423 (19.1)	685 (19.7)	843 (19.7)	1065 (19.4)	1417 (18.1)	1469 (17.8)
75~79	389 (23.8)	304 (22.0)	542 (24.5)	833 (23.9)	1065 (24.9)	1321 (24.0)	11,825 (23.4)	1865 (22.7)
80~84	301 (18.4)	294 (21.2)	502 (22.7)	742 (21.3)	887 (20.7)	1189 (21.6)	1731 (22.2)	1818 (22.1)
≥85	170 (10.4)	157 (11.3)	280 (12.6)	508 (14.6)	695 (16.2)	992 (18.1)	1504 (19.3)	1749 (21.2)
Sex, female	1066 (65.2)	956 (69.0)	1481 (66.8)	2314 (66.4)	2894 (67.6)	3715 (67.6)	5236 (67.0)	5517 (67.0)
Income, quintile								
<20%	170 (10.4)	132 (9.5)	136 (19.7)	746 (21.4)	872 (20.4)	1210 (22.0)	1879 (24.1)	2045 (24.8)
20~40%	131 (8.0)	114 (8.2)	152 (6.9)	259 (7.4)	326 (7.6)	409 (7.4)	672 (8.6)	712 (8.7)
40~60%	174 (10.7)	159 (11.5)	259 (11.7)	365 (10.5)	448 (10.5)	568 (10.3)	823 (10.5)	885 (10.8)
60~80%	358 (21.9)	277 (20.0)	367 (16.6)	664 (19.1)	813 (19.0)	940 (17.1)	1348 (17.3)	1363 (16.6)
≥80%	801 (49.0)	703 (50.8)	1002 (45.2)	1450 (41.6)	1821 (42.6)	2369 (43.1)	3088 (39.5)	3228 (39.2)
Comorbidities								
Diabetes	102 (6.2)	91 (6.6)	189 (8.5)	296 (8.5)	376 (8.8)	485 (8.8)	710 (9.1)	690 (8.4)
COPD	6 (0.4)	3 (0.2)	7 (0.3)	9 (0.3)	11 (0.3)	18 (0.3)	28 (0.4)	24 (0.3)
CKD	4 (0.2)	1 (0.1)	3 (0.1)	11 (0.3)	13 (0.3)	19 (0.3)	37 (0.5)	34 (0.4)
Dyslipidemia	87 (5.3)	80 (5.8)	153 (6.9)	250 (7.2)	362 (8.5)	422 (7.7)	604 (7.7)	688 (8.4)
Stroke	188 (11.5)	99 (7.2)	371 (16.7)	826 (23.7)	1064 (24.9)	1352 (24.6)	1767 (22.6)	1621 (19.7)
Hypertension	374 (22.9)	264 (19.1)	527 (23.8)	907 (26.0)	1086 (25.4)	1381 (25.1)	1887 (24.2)	1889 (22.9)
Depression	292 (17.9)	313 (22.6)	410 (18.5)	600 (17.2)	823 (17.1)	821 (14.9)	1105 (14.2)	1138 (13.8)
Antidementia medication								
Donepezil	1015 (62.1)	937 (67.7)	1545 (69.7)	2360 (67.7)	2831 (66.1)	3535 (64.3)	5215 (66.8)	5700 (69.2)
Galantamine	498 (30.5)	338 (24.4)	390 (17.6)	508 (14.6)	467 (10.9)	624 (11.4)	790 (10.1)	778 (9.5)
Rivastigmine	121 (7.4)	72 (5.2)	60 (2.7)	173 (5.0)	293 (6.9)	385 (7.0)	951 (12.2)	891 (10.8)
Memantine	0 (0)	38 (2.7)	221 (10.0)	443 (12.7)	689 (16.1)	952 (17.3)	854 (10.9)	864 (10.5)

Data are presented as number (%), unless otherwise indicated.

**Table 3:** Characteristics of dementia patients according to year of diagnosis (2011~2018).

	2011	2012	2013	2014	2015	2016	2017	2018
Total number	9248 (100)	8935 (100)	9271 (100)	9625 (100)	9553 (100)	9403 (100)	9206 (100)	8817 (100)
Age								
60~64	520 (5.6)	531 (5.9)	523 (5.6)	550 (5.7)	602 (6.3)	574 (6.1)	577 (6.3)	510 (5.8)
65~69	742 (8.0)	754 (8.4)	717 (7.7)	702 (7.3)	659 (6.9)	621 (6.6)	601 (6.5)	549 (6.2)
70~74	1637 (17.7)	1477 (16.5)	1465 (15.8)	1471 (15.3)	1330 (13.9)	1107 (11.8)	1054 (11.5)	983 (11.2)
75~79	2112 (22.8)	2118 (23.7)	2300 (24.8)	2251 (23.4)	2216 (23.2)	2235 (23.8)	2031 (22.1)	1975 (22.4)
80~84	2107 (22.7)	2069 (23.2)	2056 (22.2)	2250 (23.4)	2374 (24.9)	2421 (25.8)	2422 (26.3)	2266 (25.7)
≥85	2130 (23.0)	1986 (22.2)	2210 (23.8)	2401 (25.0)	2372 (24.8)	2445 (26.0)	2521 (27.4)	2534 (28.7)
Sex, female	6219 (67.3)	5961 (66.7)	6047 (65.2)	6326 (65.7)	6129 (64.2)	6017 (64.0)	5807 (63.1)	5529 (62.7)
Income, quintile								
<20%	2387 (25.8)	2122 (23.8)	2264 (24.4)	2387 (24.8)	2309 (24.2)	2274 (24.2)	2285 (27.8)	2160 (24.5)
20~40%	841 (9.1)	875 (9.8)	819 (8.8)	975 (10.1)	929 (9.7)	911 (9.7)	952 (10.3)	894 (10.1)
40~60%	946 (10.2)	898 (10.1)	994 (10.7)	1018 (10.6)	997 (10.4)	1028 (10.9)	1071 (11.6)	975 (11.1)
60~80%	1547 (16.7)	1515 (17.0)	1552 (16.7)	1641 (17.1)	1593 (16.7)	1606 (17.08)	1528 (16.6)	1486 (16.9)
≥80%	3527 (38.1)	3525 (39.5)	3642 (39.3)	3604 (37.4)	3725 (39.0)	3584 (38.1)	3370 (36.6)	3302 (37.5)
Comorbidities								
Diabetes	784 (8.5)	700 (7.8)	717 (7.7)	767 (8.0)	764 (8.0)	706 (7.5)	633 (6.9)	639 (7.3)
COPD	38 (0.4)	43 (0.5)	42 (0.5)	24 (0.25)	25 (0.3)	15 (0.2)	21 (0.2)	20 (0.2)
CKD	36 (0.4)	47 (0.5)	54 (0.6)	69 (0.7)	60 (0.6)	50 (0.5)	55 (0.6)	52 (0.6)
Dyslipidemia	826 (8.9)	756 (8.5)	872 (9.4)	903 (9.4)	927 (9.7)	982 (10.4)	1006 (10.9)	939 (10.7)
Stroke	1716 (18.6)	1646 (18.4)	1611 (17.4)	1648 (17.1)	7565 (16.4)	1549 (16.5)	1398 (15.2)	1302 (14.8)
Hypertension	1862 (20.1)	1694 (19.0)	1690 (18.2)	1749 (18.2)	1687 (17.7)	1585 (16.9)	1493 (16.2)	1328 (15.1)
Depression	1271 (13.7)	1214 (13.6)	1303 (14.1)	1344 (14.0)	1353 (14.2)	1345 (14.3)	1461 (15.9)	1526 (17.3)
Antidementia medication								
Donepezil	6554 (70.9)	6717 (75.2)	7188 (77.5)	7437 (77.3)	7636 (79.9)	7751 (82.4)	7643 (83.0)	7321 (83.0)
Galantamine	644 (7.0)	415 (4.6)	300 (3.2)	305 (3.2)	257 (2.7)	276 (2.9)	192 (2.1)	151 (1.7)
Rivastigmine	1090 (11.8)	1151 (12.9)	1167 (12.6)	1208 (12.6)	922 (9.7)	700 (7.4)	639 (6.9)	663 (7.5)
Memantine	960 (10.4)	652 (7.3)	616 (6.6)	675 (7.0)	738 (7.7)	675 (7.2)	732 (8.0)	682 (7.7)

Data are presented as number (%), unless otherwise indicated.

## Data Availability

The datasets generated or analyzed during the current study are available from the National Health Insurance Sharing Service (NHIS) at https://nhiss.nhis.or.kr (accessed on 15 December 2021). Upon an individual researcher’s dataset request, NHIS provides customized data to the researcher.

## Supplementary Materials

The following supporting information can be downloaded at: https://www.mdpi.com/article/10.3390/jcm11030819/s1, Table S1: Pharmaceutical prescription codes for antidementia drugs in Korea. Table S2: Crude incidence of dementia stratified by age and sex from 2003 to 2018. Data are presented as per 100,000 population at risk (95% CI). Table S3: Comparing characteristics of patients with incident dementia before and after 2008.
