# Supplementary material for: Annual Incidence of Dementia from 2003 to 2018 in Metropolitan Seoul, Korea: A Population-Based Study
Source: J Clin Med. 2022 Feb 3;11(3):819. doi: 10.3390/jcm11030819 (PMC8836574; doi:10.3390/jcm11030819)
Supplement: Supplementary file 1 [file jcm-11-00819-s001.zip › í+Suppl Table S1.pdf]

Table S1. Pharmaceutical prescription codes for antidementia drugs.

Antidementia drug	Drug classification code	Pharmaceutical prescription codes
Donepezil	119	148601ATB, 148601ATD, 148602ATB, 148602ATD, 148603ATB, 643401ATD, 643402ATD
Rivastigmine	119	224501ACH, 224503ACH, 224504ACH, 224505ACH, 224506CPC, 224507CPC, 224508CPC
Galantamine	119	385203ACR, 385203ATR, 385204ACR, 385204ATR, 385205ACR, 385205ATR
Mematine	119	190001ALQ, 190001ATB, 190002ASY, 190030ASY, 190031ALQ, 190003ATD, 190004ATD, 190004ATB
