# Supplementary material for: Annual Incidence of Dementia from 2003 to 2018 in Metropolitan Seoul, Korea: A Population-Based Study
Source: J Clin Med. 2022 Feb 3;11(3):819. doi: 10.3390/jcm11030819 (PMC8836574; doi:10.3390/jcm11030819)
Supplement: Supplementary file 1 [file jcm-11-00819-s001.zip › í+Suppl Table S2.pdf]

Table S2 Crude incidence of dementia stratified by age and sex.

	Total year	2003	2004	2005	2006	2007	2008	2009	2010	2011	2012	2013	2014	2015	2016	2017	2018
Total incidence rate	457.23(454.51 - 459.95)	160.27(152.50 - 168.04)	129.82(122.98 - 136.66)	199.66(191.35 - 207.98)	304.53(294.42 - 314.65)	305.95(296.78 - 315.11)	373.43(363.56 - 383.31)	504.74(493.54 - 515.93)	587.32(574.64 - 600.01)	641.51(628.44 - 654.59)	588.76(576.55 - 600.97)	586.72(574.78 - 598.67)	581.96(570.33 - 593.59)	551.03(539.98 - 562.08)	520.72(510.19 - 531.25)	486.79(476.84 - 496.73)	448.26(438.90 - 457.61)
Age groups																	
60~64	82.01(80.05-83.98)	40.29(34.15-46.44)	35.11(29.41-40.80)	40.19(34.13-46.25)	62.09(54.56-69.63)	51.31(44.82-57.81)	67.80(60.49-75.10)	96.96(88.42-105.50)	98.90(90.05-107.75)	107.57(98.33-116.82)	106.05(97.03-115.07)	102.59(93.80-111.39)	103.18(94.56-111.80)	104.83(94.46-113.20)	93.16(85.54-100.78)	90.56(83.17-97.95)	76.67(70.02-83.32)
65~69	154.42(151.30-157.55)	94.55(82.83-106.27)	71.87(62.03-81.71)	100.69(89.29-112.08)	145.25(131.90-158.59)	138.79(127.19-150.39)	146.12(134.41-157.83)	201.58(187.94-215.33)	228.91(213.54-244.29)	197.42(183.21-211.62)	197.19(183.11-211.26)	179.27(166.15-192.39)	168.31(155.86-180.76)	152.93(141.26-164.61)	145.06(133.65-156.46)	135.49(124.66-146.32)	121.87(111.68-132.07)
70~74	396.59(390.64-402.54)	232.36(208.33-256.40)	168.97(149.14-188.80)	234.36(212.03-256.70)	354.94(328.36-381.53)	335.37(312.73-358.01)	393.97(370.31-417.63)	486.06(460.75-511.37)	572.89(543.59-602.19)	597.07(568.14-625.99)	486.18(461.39-510.98)	464.70(440.90-488.50)	458.65(435.21-482.09)	412.59(390.42-434.77)	340.43(320.38-360.49)	317.73(298.55-336.91)	283.49(265.77-301.21)
75~79	927.32(915.	406.75(366.	297.49(264.	501.24(459.	744.04(693.	723.83(680.	841.97(796.	1,073.90(1,0	1,243.25(1,1	1,294.31(1,2	1,203.79(1,1	1,211.92(1,1	1,098.26(4,0	1,037.35(99	975.02(934.	797.52(762.	748.47(715.

	91-938.73)	33-447.17)	05-330.93)	05-543.44)	51-794.57)	36-767.30)	57-887.37)	24.63-1,123.17)	86.83-1,299.68)	39.11-1,349.51)	52.52-1,255.05)	62.39-1,261.45)	52.89-1,143.63)	4.16-1,080.54)	60-1,015.45)	83-832.20)	46-781.48)
80~84	1,691.90(1,671.11-1,712.70)	506.57(449.34-563.80)	477.91(423.28-532.54)	790.81(721.63-859.99)	1,158.05(1,074.73-1,241.38)	1,080.34(1,009.24-1,151.44)	1,362.45(1,285.01-1,439.90)	1,806.42(1,721.32-1,891.52)	2,206.12(2,104.71-2,307.54)	2,433.78(2,329.86-2,537.71)	2,242.33(2,145.71-2,338.95)	2,100.40(2,009.61-2,191.19)	2,139.07(2,050.68-2,227.46)	2,054.33(1,971.69-2,136.97)	1,925.1(1,848.41-2,001.78)	1,776.17(1,705.43-1,846.91)	1,538.44(1,475.10-1,601.79)
≥ 85	2,496.09(2,464.93-2,527.25)	471.36(400.50-542.22)	425.03(358.54-491.51)	712.23(628.81-795.66)	1,226.05(1,119.43-1,332.66)	1,279.01(1,183.92-1,374.10)	1,659.81(1,556.52-1,763.10)	2,356.48(2,237.39-2,475.58)	3,156.01(3,008.10-3,303.93)	3,645.89(3,491.05-3,800.72)	3,177.70(3,037.94-3,317.46)	3,275.77(3,139.20-3,412.35)	3,294.68(3,162.90-3,426.47)	3,082.44(2,958.39-3,206.49)	3,005.90(2,886.75-3,125.05)	2,875.42(2,763.18-2,987.67)	2,713.61(2,607.96-2,819.27)
Male only																	
Total incidence rate of male	344.84(341.35 - 348.34)	123.99(113.80 - 134.19)	88.93(80.52 - 97.35)	145.77(135.23 - 156.31)	225.17(212.26 - 238.07)	217.09(205.66 - 228.52)	264.73(252.43 - 277.02)	363.79(349.74 - 377.84)	423.75(407.81 - 439.69)	459.28(442.92 - 475.63)	427.89(412.51 - 443.26)	445.49(430.11 - 460.87)	435.28(420.42 - 450.13)	431.52(417.06 - 445.97)	410.27(396.45 - 424.09)	393.50(380.27 - 406.73)	366.35(353.83 - 378.87)
60~64	88.11(85.18-91.04)	33.80(25.89-41.72)	24.34(17.66-31.02)	33.61(25.79-41.42)	62.59(51.87-73.31)	54.44(44.90-63.99)	64.03(53.88-74.17)	103.31(90.68-115.94)	102.29(89.37-115.20)	113.50(99.86-127.14)	106.47(93.48-119.46)	118.83(105.20-132.46)	120.11(106.70-133.53)	125.83(112.55-139.11)	109.93(97.93-121.94)	107.58(95.89-119.28)	85.10(74.91-95.29)

65~69	149.73(145.30-154.15)	79.25(63.72-94.79)	63.07(49.82-76.32)	85.82(70.78-100.87)	121.00(103.62-138.40)	119.34(104.05-134.64)	132.18(116.33-148.04)	188.00(169.22-206.78)	216.72(195.37-238.06)	199.35(178.90-219.81)	190.32(170.38-210.26)	174.69(155.98-193.40)	164.67(146.85-182.50)	152.67(135.76-169.59)	157.11(139.87-174.35)	148.30(131.84-164.75)	139.70(123.81-155.59)
70~74	356.95(348.66-365.25)	217.52(181.23-253.81)	125.09(98.80-151.37)	200.85(169.43-232.27)	296.90(260.25-333.56)	269.29(239.12-299.46)	307.65(276.80-338.51)	396.03(362.50-429.56)	494.99(455.18-534.79)	547.25(507.00-587.51)	421.95(388.57-455.33)	434.15(400.90-467.39)	414.43(382.19-446.66)	379.28(348.47-410.09)	321.79(293.42-350.17)	325.45(297.04-353.86)	287.20(261.04-313.36)
75~79	790.34(774.06-806.63)	377.03(310.66-443.39)	221.87(172.94-270.79)	412.24(347.96-476.52)	656.70(577.48-735.92)	557.10(495.29-618.92)	692.01(626.07-757.96)	879.96(809.49-950.43)	1017.24(937.10-1097.38)	993.33(918.40-1068.27)	946.28(876.58-1015.99)	1003.97(935.51-1072.44)	929.08(866.25-991.91)	938.73(877.26-1000.20)	836.61(780.93-892.30)	694.84(646.96-742.72)	702.86(655.55-750.17)
80~84	1,393.19(1,361.56-1,424.83)	504.55(404.14-604.96)	423.50(333.47-513.53)	678.50(565.29-791.71)	1034.33(894.43-1174.23)	850.63(741.64-959.62)	1085.54(965.85-1205.23)	1333.08(1206.34-1459.82)	1750.61(1591.32-1909.90)	1927.34(1765.97-2088.71)	1866.10(1715.14-2017.06)	1651.13(1515.82-1786.44)	1760.97(1628.22-1893.72)	1734.92(1611.58-1858.27)	1604.78(1492.66-1716.90)	1469.59(1367.62-1571.55)	1260.79(1170.79-1350.78)
≥ 85	2,119.23(2,065.12-2,173.34)	420.44(288.48-552.40)	413.48(285.34-541.62)	822.18(648.41-995.95)	1131.10(934.38-1327.82)	1118.85(953.08-1284.63)	1378.68(1204.25-1553.10)	1988.99(1786.86-2191.11)	2635.89(2378.22-2893.57)	2918.29(2653.04-3183.54)	2683.81(2437.75-2929.88)	2955.24(2706.90-3203.58)	2525.28(2306.54-2744.02)	2445.69(2237.08-2654.30)	2576.05(2369.41-2782.70)	2531.01(2336.58-2725.43)	2324.92(2147.28-2502.57)
Female only																	

Total incidence rate of female	551.59(547.54 - 555.64)	189.87(178.48 - 201.27)	163.567(153.20 - 173.94)	244.53(232.08 - 256.98)	370.58(355.48 - 385.68)	380.55(366.69 - 394.42)	464.96(450.01 - 479.92)	623.50(606.61 - 640.38)	725.12(705.99 - 744.26)	795.18(775.42 - 814.95)	724.70(706.30 - 743.10)	706.07(688.27 - 723.86)	706.04(688.64 - 723.44)	651.90(635.58 - 668.22)	613.69(598.18 - 629.19)	565.22(550.68 - 579.76)	516.99(503.36 - 530.62)
60~64	76.26(73.61-78.90)	46.94(37.50-56.37)	46.05(36.79-55.31)	46.84(37.56-56.11)	61.60(51.01-72.19)	48.30(39.47-57.12)	71.41(60.92-81.90)	90.93(79.38-102.48)	95.71(83.57-107.84)	101.99(89.45-114.54)	105.66(93.12-118.19)	87.48(76.20-98.77)	87.48(76.46-98.51)	85.64(75.17-96.11)	77.96(68.33-87.58)	75.21(65.92-84.49)	69.09(60.39-77.80)
65~69	158.81(154.41-163.22)	108.51(91.15-125.88)	80.11(65.65-94.56)	114.91(97.88-131.93)	168.60(148.45-188.75)	157.84(140.43-175.24)	159.70(142.49-176.90)	214.77(195.00-234.55)	240.68(218.59-262.77)	195.58(175.83-215.32)	203.55(183.70-223.40)	183.48(165.08-201.88)	171.63(154.23-189.02)	153.17(137.03-169.31)	134.18(119.05-149.31)	123.93(109.64-138.22)	105.94(92.86-119.02)
70~74	430.74(422.28-439.20)	242.71(210.71-274.70)	200.90(172.48-229.32)	259.96(228.72-291.21)	400.51(36.79-438.24)	389.89(356.91-422.87)	467.29(432.25-502.34)	564.08(526.83-601.33)	641.42(598.92-683.92)	641.81(600.50-683.13)	545.23(508.86-581.61)	492.74(458.81-526.68)	499.10(465.27-532.94)	442.86(411.12-474.59)	357.10(328.83-385.37)	310.97(284.98-336.96)	280.26(256.17-304.35)
75~79	1,025.78(1,010.05-1,041.51)	422.32(371.48-473.17)	337.93(293.77-382.09)	550.11(495.09-605.13)	792.98(727.82-858.13)	826.29(767.27-885.31)	937.68(876.35-999.01)	1,203.49(1,136.13-1,270.86)	1,397.50(1,319.91-1,475.10)	1,509.09(1,431.06-1,587.12)	1,394.30(1,321.52-1,467.08)	1,371.06(1,301.07-1,441.06)	1,231.79(1,167.51-1,296.06)	1,117.01(1,056.75-1,177.27)	1,089.23(1,031.51-1,146.95)	884.00(834.44-933.57)	786.88(740.94-832.83)
80~84	1,856.91(1,829.77-	507.54(437.89-	504.26(435.89-	843.76(757.08-	1,215.44(1,112.16-	1,196.08(1,104.34-	1,501.06(1,401.48-	2,042.41(1,931.64-	2,422.10(2,293.08-	2,681.46(2,548.35-	2,436.94(2,312.87-	2,346.50(2,227.12-	2,356.36(2,239.95-	2,249.32(2,139.58-	2,129.85(2,026.58-	1,979.04(1,882.79-	1,728.24(1,641.12-

	1,884.05)	577.19)	572.62)	930.45)	1,318.73)	1,287.82)	1,600.63)	2,153.18)	2,551.11)	2,814.57)	2,561.01)	2,465.89)	2,472.77)	2,359.06)	2,233.12)	2,075.29)	1,815.35)
≥ 85	2,643.76(2,605.93-2,681.59)	488.99(405.25-572.73)	429.12(351.36-506.88)	672.37(577.76-766.99)	1,261.34(1,134.68-1,388.00)	1,343.74(1,228.24-1,459.24)	1,775.34(1,648.45-1,902.23)	2,508.81(2,362.66-2,654.96)	3,353.50(3,174.41-3,532.59)	3,918.75(3,730.52-4,106.99)	3,362.66(3,194.10-3,531.21)	3,396.05(3,232.97-3,559.13)	3,591.25(3,429.30-3,753.21)	3,330.74(3,178.72-3,482.77)	3,177.17(3,032.31-3,322.03)	3,018.42(2,881.61-3,155.23)	2,882.65(2,752-20-3,013.10)

Data are presented as per 100,000 population at risk (95% CI).
