# Supplementary material for: Annual Incidence of Dementia from 2003 to 2018 in Metropolitan Seoul, Korea: A Population-Based Study
Source: J Clin Med. 2022 Feb 3;11(3):819. doi: 10.3390/jcm11030819 (PMC8836574; doi:10.3390/jcm11030819)
Supplement: Supplementary file 1 [file jcm-11-00819-s001.zip › í+Suppl Table S3.pdf]

Table S3 Comparing characteristics of patients with incident dementia before and after 2008.

	Diagnosed with dementia before 2008	Diagnosed with dementia after 2008	*P*
Year of diagnosis	2005-2007	2009-2011.	
Total number	9,980 (100)	25,291 (100)	<0.001
Dementia incidence per 10^5 person-year (95% CI)	273.21 (267.85-278.57)	576.01 (568.91-583.11)	
Age			<0.001
60~64	670 (6.71)	1,495 (5.91)	
65~69	1,305 (13.08)	2,432 (9.62)	
70~74	1,951 (19.55)	4,523 (17.88)	
75~79	2,440 (24.45)	5,802 (22.94)	
80~84	2,131 (21.35)	5,656 (22.36)	
≥ 85	1,483 (14.86)	5,383 (21.29)	
Sex, female	6,689 (67.02)	16,972 (67.11)	<0.001
Income, quintile			<0.001
< 20%	2,054 (20.58)	6,311(24.95)	
20~40%	737 (7.38)	2,225 (8.8)	
40~60%	1,072 (10.74)	2,654 (10.49)	
60~80%	1,844 (18.48)	4,258 (16.84)	
≥ 80%	4,273 (42.82)	9,843 (38.92)	
Comorbidities			
Diabetes	561 (5.62)	2,046 (8.09)	<0.001
Chronic kidney disease	27 (0.27)	107 (0.42)	0.036
Dyslipidemia	765 (7.67)	2,118 (8.37)	0.029
Stroke	1233 (12.35)	2,549 (10.08)	<0.0001
Hypertension	2,520 (25.25)	5,638 (22.29)	<0.0001
Depression	1,743 (17.46)	3,514 (13.89)	<0.0001
Connective tissue disorder	28 (0.28)	19 (0.08)	<0.0001
Peptic ulcer	70 (0.7)	12 (0.05)	<0.0001
Congestive Heart Failure	8 (0.08)	2 (0.01)	0.001
Peripheral vascular disease	145 (1.45)	297 (1.17)	0.038
Antidementia medication			<0.001
Donepezil	6,736 (67.49)	17,469 (69.07)	
Galantamine	1,365 (13.68)	2,212 (8.75)	
Rivastigmine	526 (5.27)	2,932 (11.59)	
Memantine	1,353 (13.56)	2,678 (10.59)	

Data are presented as number (%), unless otherwise indicated
